# Cathepsin S Signals via PAR2 and Generates a Novel Tethered Ligand Receptor Agonist

**DOI:** 10.1371/journal.pone.0099702

**Published:** 2014-06-25

**Authors:** Sarina B. Elmariah, Vemuri B. Reddy, Ethan A. Lerner

**Affiliations:** Cutaneous Biology Research Center, Department of Dermatology, Massachusetts General Hospital and Harvard Medical School, Boston, Massachusetts, United States of America; University Paris Sud, France

## Abstract

Protease-activated receptor-2 is widely expressed in mammalian epithelial, immune and neural tissues. Cleavage of PAR2 by serine proteases leads to self-activation of the receptor by the tethered ligand SLIGRL. The contribution of other classes of proteases to PAR activation has not been studied in detail. Cathepsin S is a widely expressed cysteine protease that is upregulated in inflammatory conditions. It has been suggested that cathepsin S activates PAR2. However, cathepsin S activation of PAR2 has not been demonstrated directly nor has the potential mechanism of activation been identified. We show that cathepsin S cleaves near the N-terminus of PAR2 to expose a novel tethered ligand, KVDGTS. The hexapeptide KVDGTS generates downstream signaling events specific to PAR2 but is weaker than SLIGRL. Mutation of the cathepsin S cleavage site prevents receptor activation by the protease while KVDGTS retains activity. In conclusion, the range of actions previously ascribed to cysteine cathepsins in general, and cathepsin S in particular, should be expanded to include molecular signaling. Such signaling may link together observations that had been attributed previously to PAR2 or cathepsin S individually. These interactions may contribute to inflammation.

## Introduction

Protease-activated receptors (PARs) comprise a family of G-protein-coupled receptors that are self-activated following proteolytic cleavage of their extracellular N-terminal domains. The PAR family consists of four members, PAR1, PAR2, PAR3 and PAR4, that are expressed in epithelial, immune, neural and vascular tissues and have been implicated in inflammation, pain, itch, hemostasis, thrombosis and carcinogenesis[1], [2]. Given their wide distribution and involvement in numerous physiologic and pathophysiologic conditions, the importance of understanding how these receptors are activated and subsequently regulated, and whether they can be targeted for therapeutic purposes, continues to grow.

PAR2 signaling has been implicated in the development of inflammatory and bowel diseases, asthma, itch and pain. The receptor is expressed on many types of cells including keratinocytes, sensory neurons, fibroblasts, endothelial cells, and inflammatory cells[2], [3]. Consistent with this broad expression, PAR2 is involved in numerous physiologic and pathophysiologic processes in response to both endogenous and exogenous proteases. PAR2 has been shown to be activated by several families of proteases expressed in the skin, including serine proteases (e.g. kallikreins 5 and 7, mast cell tryptase) and the cysteine protease, cathepsin S[4], [5]. PAR2 may also act as a sensor for exogenous protease activity, with *in vitro* data supporting activation by a cockroach allergen and the dust mite allergen der p 1[6], gingipain from a gingivitis-inducing bacterium[7], and the plant proteases mucunain, papain, bromelain and ficin amongst others[8].

Proteases cleave the extracellular N-terminal domain of PARs. This cleavage generates a new N-terminus and unmasks a tethered ligand that triggers intracellular signaling[9], [10]. Synthetic hexapeptides corresponding to the first six amino acids of the newly formed N-terminus can serve as ligands for PARs. This action is independent of receptor cleavage[11]–[13] although micromolar concentrations of hexapeptide are needed for activity. It has been suggested that synthetic hexapeptides or different proteases acting on the same PAR may stimulate different downstream signaling pathways[14] but little is known about the specific cleavage sites and resultant ligands produced by different proteases.

To better understand the mechanisms of PAR2 activation and regulation, we sought to identify cleavage sites and downstream signaling events induced by an endogenous cysteine protease, cathepsin S. Cysteine cathepsins, including cathepsin S, have been broadly implicated in health and disease including inflammation, cardiovascular and pulmonary disease, obsesity, itch and pain[15]. Cathepsin S is unique among cysteine proteases in that its expression is up-regulated by IFN-γ, which supports a role in inflammation[16]. Cathepsin S is expressed in many types of cells. It has a broad pH profile. It is active at neutral pH, consistent with extracellular biological activity[16]. Cathepsin S is a potent elastase and is critical to antigen presentation as it cleaves the Ii chain[17]–[19]. Cathepsin S causes pain via a PAR2-dependent mechanism in a mouse model of inflammatory bowel disease and evokes itch when applied to human skin, potentially as a result of PAR2 activation[5], [20]. These observations support the possibility that cathepsin S cleaves PAR2 but the cleavage site(s) involved in activation of this receptor have not been identified.

We demonstate that cathepsin S cleaves the N-terminus of PAR2 at a site distinct from that of serine proteases. Substitution of a single residue near the N-terminus prevents cathepsin S from activating the receptor. KVDGTS, a hexapeptide derived from the newly exposed N-terminal sequence, retains activity on the wildtype and mutant receptors. Cathepsin S and KVDGTS activate downstream signaling in keratinocytes and a heterologous cell line. The results presented here reveal that cathepsin S can participate in signal transduction via activation of PAR2.

## Materials and Methods

### Cell culture

HeLa cells were obtained from the ATCC and maintained in DMEM supplemented with fetal bovine serum (Fisher Biochemicals), L-glutamine, penicillin and streptomycin. Normal human epidermal keratinocytes (NHEKs) were harvested from neonatal foreskin and maintained in Keratinocyte Serum-Free media supplemented with epidermal growth factor, bovine pituitary extract, L-glutamine, penicillin and streptomycin. All reagents were obtained from Invitrogen unless otherwise noted.

### PARs, Peptides and proteases

cDNA for human PAR2 was obtained commercially and cloned into pcDNA3.1(−). The superscript number following the first amino acid of the peptides corresponds to the position in full length PAR2. Several peptides were synthesized by Peptide 2.0 (Chantilly, VA): G^28^TNRSSKGRSLIGKVDGTSHVTGKGVT represents 27 N-terminal residues of PAR2 following processing. K^41^VDGTS and I^39^GKVDG are PAR2 cleavage peptides and S^46^HVTGK is a downstream control peptide. The conventional serine protease tethered ligand hexapeptide SLIGRL served as a positive control and the reverse peptide, LRGILS as a negative control. SLIGRL is derived from the sequence of murine PAR2 but is used as opposed to SLIGKV in both human and mouse studies of PARs. S^37^LIGKVDGTS and the KVDGTS reverse peptide, STGDVK, were synthesized by GenScript (Piscataway, NJ).

Recombinant human cathepsin S was produced as follows. The human cathepsin S proprotein coding region was isolated by PCR using the forward and reverse primers GCGCCATGGTGGCACAGTTGCATAAAGATCCTAC CCTG and GCGCTCGAGCTAGATTTCTGGGTAAGAGGGAAAGCTAG and a template of plasmid DNA containing the entire coding sequence of human cathepsin S. The PCR product was cut with Nco I and Xho I, and cloned into the pTYB4 expression vector from New England Biolabs for expression in *E.coli*. Cells were grown in LB medium induced with 0.3 mM IPTG, harvested by centrifugation, resuspended in lysis buffer A (0.5 M NaCl, 0.05 M Tris, 5 mM EDTA, pH 8.0) and lysed in a French press. The lysate was centrifuged and the protein was found predominantly in inclusion bodies which were dissolved in buffer B (10 mM DTT and 8 M urea), and refolded in the presence of buffer B containing 0.7 M L-arginine, 0.01 M glutathione, 0.001 M glutathione disulfide, and 0.005 M EDTA as described[21]. The refolded procathepsin S was concentrated by Minimate Concentrator (Pall Corporation) and washed three times with buffer A. It was activated after adjusting to pH 4.5 with acetic acid, adding DTT to 5 µM and incubating at 37°C for 3 hours. Recombinant cathepsin S was purified using Centricon Plus-70 membranes (Millipore) with a MW cut off of 10 kDa, dialyzed against buffer A and aliquoted and stored at −80°C. SDS-PAGE was used to confirm the presence of pro and activated human cathepsin S based on predicted MW. The specific activity of cathepsin S was assayed using Z-VVR-AMC in fluorimetric and Z-FR-pNA in colorimetric assays[22] and determined to be 10,000 mU/mg protein.

### Cathepsin S digestion of the human PAR2 N-terminal peptide

A peptide comprising the N-terminus of PAR2, GTNRSSKGRSLIGKVDGTSHVTGKGVT, was dissolved in 50 mM Tris, pH 7.0, 0.1 M NaCl, 0.1 mM EDTA, and 0.01% Tween-20 to a concentration of 100 mM. 100 µL of this solution was incubated with cathepsin S (1 µM final concentration) at 37°C for 20 minutes. Acetonitrile and formic acid were added to final concentrations of 2% and 0.1%, respectively, and the material was immediately frozen at −80°C. The reaction samples were analyzed by the Harvard Microchemistry & Proteomics Analysis Facility (Cambridge, MA). Label-free quantitation based on spectral counting has been shown to be a reliable technique. After digestion with a proteolytic enzyme (trypsin or others) the number of peptide-spectrum matches (PSMs) observed during an LCMS analysis correlates well with relative protein abundances in the sample[23], [24].

The LC/MS/MS system was configured with a nanoAcquity UPLC (Waters, Milford MA) running 0.1% formic acid in water (A-buffer) and 0.1% formic acid in acetonitrile (B-buffer) using self-packed glass capillary 100 µ ID Integrafrit trapping column and 75 µ ID Picofrit analytical columns (New Objective, Woburn MA) packed with 5 cm of 5 u Magic C18AQ and 20 cm of 3 µ Magic C18AQ (Michrom Bioresources) respectively. Sample trapping was performed at 2 ul/min followed by the analytcal gradient at 0.3 µ/min. The gradient started after 15 minutes of trapping, and proceeded from 2%B to 52%B/10 minutes.

The mass spectrometer used was a LTQ-Orbitrap XL (Thermo Fisher Scientific, San Jose, CA) with full MS acquired in profile mode in the Orbitrap at 60,000 resolution across the mass range of 395–1600 Da, using a common background ion as an internal calibrant (445.120025 Da, polycyclosiloxane). From the full scan, the top 4 ions by intensity were selected for MS/MS in the linear ion trap with a relative collision energy of 35%. Samples were diluted 1∶10 with 0.1% formic acid in water (A-buffer) and 1 µl injected onto the LC/MS/MS system. Tandem mass spectra were searched with SEQUEST against the Uniprot Sprot database followed by direct searching of the sequences of PAR2. Data processing was performed in a custom version of Proteomics Browser Suite (PBS) v2.8 (ThermoFisher Scientific). PSMs were accepted with a mass tolerance of <1 ppm and score threshold to meet an estimated false discovery rate of <1% using a reverse decoy database strategy. Sf for each peptide is a single score calculated in PBS by a neural network with Xcorr, DeltaCn, Sp, RSp, peptide mass, charge state, and database size as inputs.

### Calcium imaging

Human PAR2 cDNA was cloned as an Xho I-Hind III fragment (1206 bp) into pcDNA3.1(−). 10 µg of DNA were diluted into 0.5 ml of DMEM and mixed with 0.5 ml of DMEM containing 10 µl of Lipofectamine 2000 transfection reagent and incubated at room temperature for 20 minutes. HeLa cells were grown to confluence, trypsinized and 1×10^6^ cells were pelleted in a 15 ml tube by centrifugation at 1000 rpm for 5 minutes. The DMEM-Lipofectamine 2000-DNA mixture (1 ml) was added to the cell pellet, resuspended gently and incubated at room temperature for 20 minutes. Four ml of complete DMEM with 10% FBS were added to the tube, mixed by inverting the tube several times, plated into a 96-well glass bottom plate at 20,000 cells/well, and placed in a 37°C CO_2_ incubator. HeLa cells transfected with vector alone or salmon sperm DNA (shown in the figures) were plated as a control. The medium was aspirated two days after transfection. Fura-2 was used as a ratiometric calcium-sensitive indicator as follows. 100 µl of complete DMEM containing 2 µM of Fura-2 were added to each well and left at room temperature in the dark for 1 hr. Following loading with Fura-2, the medium was aspirated and replaced with 90 µl of HEPES-buffered saline (20 mM HEPES, 115 mM NaCl, 5.4 mM KCl, 2 mM CaCl_2_, 0.8 mM MgCl_2_, 13.8 mM glucose, pH 7.4).

Imaging was performed using a Zeiss Axiovert 200 M microscope platform equipped with a flipping filter wheel for ratiometric imaging. Axiovision software, version 4.6 was used for image analysis of the cells which were excited at 340 nm and 380 nm. Ten µl of peptide agonist were added at 20 seconds after the start of the excitation procedure. Images were taken every 5 seconds, including at zero time, during a 3-minute period or longer if required. The software later analyzed all images taken during each excitation period. Typically, ratiometric changes were measured in 10–20 cells in each image. An average of the fluorescence of the cells in each image was calculated and plotted against time in seconds.

### Concentration-effect measurements for peptides

HeLa cells transfected with PAR2 cDNA were subjected to ratiometric imaging as described above with PAR2 peptides, SLIGRL, KVDGTS, and SLIGKVDGTS in addition to the reverse peptides LRGILS and STGDVK at concentrations from 1 nM - 1 mM. Each of the concentration-dependent readings was performed in triplicate. Maximum intensities at each of the dilutions were calculated and plotted against concentration using GraphPad/PRISM software version 6.0 (Irvine, CA). Error bars represent +/−SEM.

### Preparation of *Gaussia* luciferase-PAR2 fusion cDNAs

*Gaussia* luciferase cDNA was cloned, without its termination codon, into pcDNA3.1(−) as an Xho I-EcoR I fragment. Human PAR2 cDNA and its mutants, M1, M2 and M3, were generated using PCR primers designed to start after the signal sequence and end with a termination codon: GCGGAATTCATCCAAGGAACCAATAGATCCTCTAAAG and GCGAAGCTTTCAATAGGAGGTCTTAACAGTGGTTGAACTTG . The PCR amplified cDNAs were cloned into EcoR I and Hind III sites C-terminal to *Gaussia* luciferase cDNA. The resulting plasmids were called pGLPAR2, pGLPAR2M1, pGLPAR2M2 and pGLPAR2M3.

### Generation of PAR2 N-terminal mutants to remove cathepsin S cleavage sites

The QuickChange site-directed mutagenesis procedure (Agilent Technologies, Santa Clara, CA) was used to generate PAR2 N-terminal mutants. PAR2M1, with a single amino acid change, was created by substituting the leucine^38^ codon, CTT, with alanine, GCT, using the primers: PAR2M1F, CTAAAGGAAGAAGCGCTATTGGTAAGGTTGATG, and PAR2M1R, CATCAACCTTACCAATAGCGCTTCTTCCTTTAG. PAR2M2 was generated by substituting the glycine^40^ codon nearest leucine^38^, GGT, with alanine, GCT, using the primers: PAR2M2F, GGAAGAAGCCCTATTGCTAAGGTTGATGGCAC and PAR2M2R, GTGCCATCAACCTTAGCAATAGGGCTTCTTCC. The PAR2M3 mutant incorporated both of these amino acid substitutions. It was generated using the primers: PAR2M3F, CTCTAAAGGAAGAAGCGCTATTGCTAAGGTTGATGGCACATC, PAR2M3R, and GATGTGCCATCAACCTTAGCAATAGCGCTTCTTCCTTTAGAG. Super coiled plasmid DNA containing human PAR2 cDNA as a Xho I-Hind III fragment in pcDNA3.1 (−) was used as the template in the PCR procedure.

### *Gaussia* luciferase assay

The plasmid DNAs pGLPAR2, pGLPAR2M1, pGLPAR2M2 and pGLPAR2M3 (10 µg of each) were transfected into 2×10^6^ HeLa cells as described above and plated into 10 cm dishes. HeLa cells transfected with salmon sperm were used as controls. Forty-eight hours after transfection, the cells were washed twice with PBS, scraped and pelleted. Cells were scraped in order to eliminate the use of trypsin, which could activate PARs, and allowed for the luminescence assay to be performed in a small volume. Pelleted cells were resuspended into 200 µl PBS, and 100 µl of each was incubated with cathepsin S at a final concentration 2 µM for 10 minutes at room temperature. Untreated cells were processed by the same methods for a negative control. Cathepsin activity was diluted by the addition of 300 µl of complete DMEM. Cells were pelleted and supernatants collected. Luminescence assays were performed in triplicate on 50 µl of the supernatants according to the instructions of the manufacturer (New England Biolabs, Ipswich, MA).

### Inositol phosphate (IP)assay

To determine the effect of PAR2 activation on Gq stimulation, we measured IP1, a downstream metabolite of IP3, using the IP-One HTRF assay from Cis Bio, Medford, MA. HeLa cells were transfected with salmon sperm DNA or PAR2 cDNA as described above, plated in 10 cm culture dishes and incubated at 37°C with 5% CO_2_. After 24 hours, the cells were trypsinized, counted, plated into 96-well plates at 80,000 cells/well and incubated for another 24 hours. The medium was replaced with a stimulation buffer containing LiCl, per the instructions of the manufacturer. Ligands, including cathepsin S (1 µM), KVDGTS (100 µM) and SLIGRL (10 µM) were added to wells and incubated at 37°C for 1 hour and IP1 levels determined. This assay was performed on multiple wells containing each ligand on three occasions. Error bars represent SEM.

### Western blot analysis for *Gaussia* luciferase

HeLa cells were transfected and treated with cathepsin S as described for the luciferase assay. After treatment with cathepsin S, cells were pelleted at 14,000 rpm for 3 minutes. Supernatants (100 µl) were collected and denatured with 1 µl of 10% SDS. Equal volumes of supernatants were prepared in loading buffer, heated at 90°C for 5 minutes, run on NuPAGE Novex Bis-Tris mini gels and transferred to nitrocellulose membranes using standard protocols. The blot was probed with a primary rabbit anti-*Gaussia* luciferase antibody. HRP-conjugated donkey anti-rabbit antibody was used to identify the relevant bands on the membrane.

### Western blot analysis for p-PKC (Ser660)

HeLa cells were transfected with PAR2 plasmid DNA as described above. Untransfected HeLa cells were plated as a control. Forty-eight hours after transfection, cells were rinsed with PBS three times. Transfected HeLa cells were incubated with either SLIGRL (10 µM), SLIGKVDGTS (10 µM), KVDGTS (100 µM), or cathepsin S (1 µM) at room temperature for 10 minutes. Untransfected HeLa cells were treated with SLIGRL (10 µM) in a similar manner. A subset of transfected HeLa cells remained untreated and served as a negative control. After incubation, cells were harvested, pelleted and lysed by sequential freeze-thaw cycles. Cell lysates were centrifuged at 4°C for 15 minutes. Supernatants were collected and protein concentrations were determined by Bradford assay. Equal amounts of cell lysates were loaded onto NuPAGE Novex Bis-Tris mini gels, electrophoresed and transferred to nitrocellulose membranes using standard protocols. The blot was first probed with rabbit anti-phospho-PKC (βII Ser660) antibody (Cell Signalling Technology, Danvers, MA), and later reprobed with a mouse anti-actin antibody to identify the control actin band at 42 kDa. Primary antibodies were labeled with HRP-conjugated donkey anti-rabbit antibody and HRP-conjugated donkey anti-mouse antibody, respectively, followed by detection of the position of the antigen on the blot.

## Results

### Cathepsin S cleaves a synthetic amino terminus of PAR2

It has been suggested previously that cathepsin S activates PAR2 but this was not demonstrated conclusively and the potential mechanism of activation was not investigated. Here we sought to determine the mechanism by which cathepsin S activates PAR signaling and asked whether, and if so at what sites, this protease cleaves the N- terminus of PAR2. Cathepsin S was incubated with a synthetic N-terminal peptide of human PAR2. Cleavage products were collected and sequenced by tandem mass spectrometry (MS/MS). Cathepsin S proteolytic activity resulted in several cleavage points (Table 1). The predominant cleavage point was after GRSL, exposing the peptide IGKVDGTSHVTGKGVT. The next most common sequence results in G at the P1 position followed by KVDGTSHVTGKGVT. Peptides based on these two sequences were studied here. Correlations with cathepsin S cleavage points in the MEROPS database and in a high-content proteomics-based profile of cathepsin S cleavage[25], including the generation of a SeqLogo, IceLogo, and HeatMap were not productive given the short sequences and thus not shown.

**Table 1:** LC/MS/MS data from cathepsin S digestion of the human PAR2 N-terminal peptide G^28^TNRSSKGRSLIGKVDGTSHVTGKGVT.

P1 position	Sequence	Counts
V	D^43^GTSHVTGKGVT	4
I	G^40^KVDGTSHVTGKGVT	3
-	G^28^TNRSSKGRSL	163
-	G^28^TNRSSKGRSLI	9
-	G^28^TNRSSKGRSLIGK	4
-	G^28^TNRSSKGRSLIGKVD	54
-	G^28^TNRSSKGRSLIGKVDGTSHVT	38
-	G^28^TNRSSKGRSLIGKVDGTSHVTG	2
-	G^28^TNRSSKGRSLIGKVDGTSHVTGKGVT	343
D	G^44^TSHVTGKGVT	8
L	I^39^GKVDGTSHVT	7
L	I^39^GKVDGTSHVTG	7
L	I^39^GKVDGTSHVTGKGVT	24
G	K^41^VDGTSHVT	13
G	K^41^VDGTSHVTG	8
G	K^41^VDGTSHVTGK	5
G	K^41^VDGTSHVTGKG	1
G	K^41^VDGTSHVTGKGV	5
G	K^41^VDGTSHVTGLGVT	29
G	R^36^SLIGKVD	4
S	S^33^KGRSLIG	4
R	S^32^SKGRSLIG	5
G	T^29^NRSSKGRSLIG	4
G	T^45^SHVTGKGVT	3
K	V^42^DGTSHVT	1
K	V^42^DGTSHVTG	2
K	V^42^DGTSHVTGKGVT	4

MS/MS analysis of cathepsin S cleavage of synthetic N-terminal PAR2. Column 1 lists the amino acid in the P1 postion. Column 2 lists the identified cleaved peptide. Column 3 lists the number of times the cleaved peptide occurred in the scan. Details of the method are provided in the Experimental Procedures section.

### PAR2 amino terminal hexapeptides activate the PAR2 receptor

PAR2 signaling is coupled primarily to Gq which leads to activation of phospholipase C (PLC), the formation of inositol triphosphate (IP3) and diacylglycerol (DAG) and calcium mobilization. PAR2 activation by the hexapeptide SLIGRL provokes transient intracellular calcium mobilization in primary keratinocytes and various cell lines. We sought to determine whether hexapeptides based on cathepsin S cleavage of the synthetic N-terminus of PAR2 could also trigger activation of the receptor.

The hexapeptides KVDGTS and IGKVDG were synthesized based on the cleavage sites produced in PAR2 following incubation with cathepsin S as indicated above. The decapeptide SLIGKVDGTS was also evaluated as it encompasses SLIGRL, IGKVDG and KVDGTS. The SLIGRL and KVDGTS reverse peptides, LRGILS and STGDVK respectively, served as controls. All of these peptides were evaluated for functionality. HeLa cells, which do not express PAR2 endogenously, were transfected with PAR2 cDNA or, as a control, salmon sperm DNA. Fura-2 calcium imaging was performed following treatment with these peptides.

**Figure 1:**
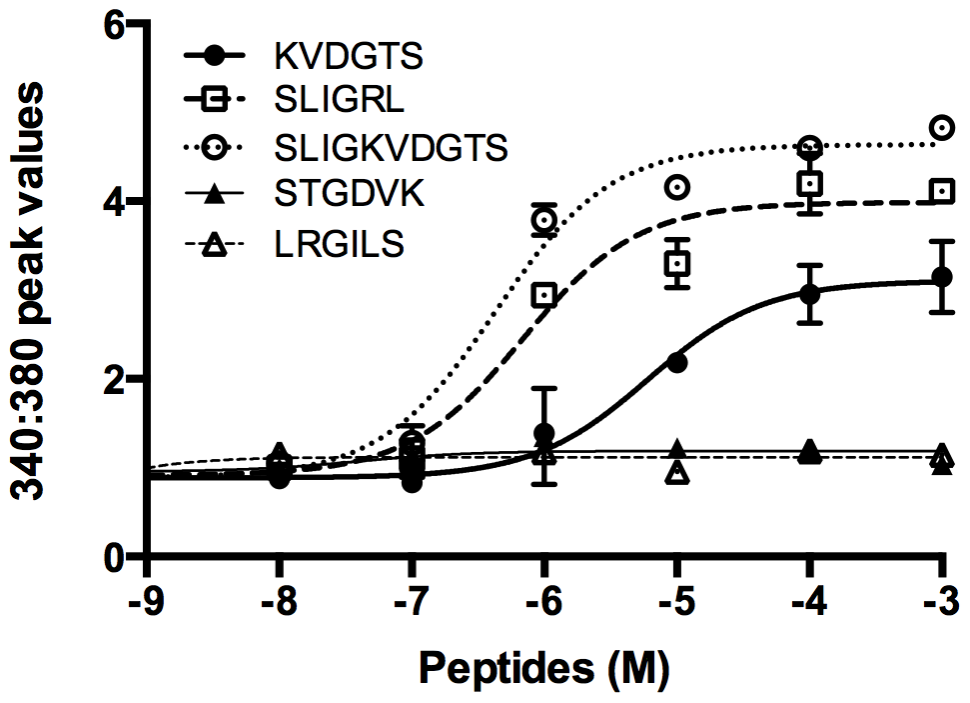
Concentration-effect curves for PAR2 derived peptides. Calcium-dependent responses were determined in PAR2-transfected HeLa cells following incubation with the indicated peptides. The curves for activating peptides are similar in shape although that for KVDGTS is shifted to the right and has a lower peak calcium response as compared to SLIGRL and SLIGKVDGTS. The inverse hexapeptides, STGDVK and LRGILS were not active.

Concentration-effect curves of the peptides on PAR2 transfected HeLa cells were generated. (Figure 1). The curves for SLIGRL and SLIGKVDGTS overlapped. The curve for KVDGTS was shifted to the right and did not achieve as high a peak response as either SLIGRL or SLIGKVDGTS. The data for IGKVDG is not included as this peptide is weaker than KVDGTS. Based on these data, we decided to use SLIGRL at 10 µM and KVDGTS at 100 µM in subsequent experiments. The reverse peptides were inactive at all concentrations examined.

**Figure 2:**
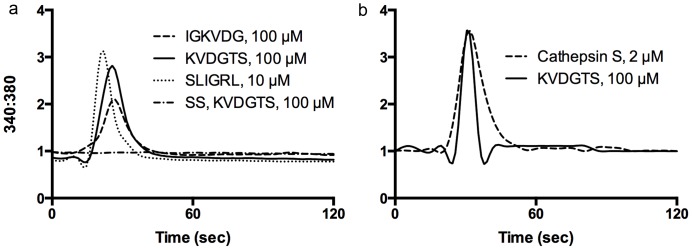
PAR2 N-terminal hexapeptides are capable of activating the receptor. a) Single-cell calcium imaging in HeLa cells transfected with PAR2 cDNA demonstrated a similar response to KVDGTS (thick solid line) and SLIGRL (thick dotted line). A weaker response was observed in response to IGKVDG (thin solid line). HeLa cells transfected with salmon sperm DNA and stimulated with KVDGTS did not respond (horizontal dotted line). b) Cathepsin S (solid line) and KVDGTS (100 µM) (dotted line) elicit similar calcium responses in NHEKs. KVDGTS (100 µM) and SLIGRL (10 µM), IGKVDG (100 µM) and cathepsin S (2 µM).

KVDGTS (100 µM) activated PAR2 with a profile similar to that for SLIGRL (10 µM) (Figure 2a) and reported previously for cathepsin S. IGKVDG (100 µM), which partially overlaps with KVDGTS, induced PAR2 activation but was approximately 50% less active than KVDGTS. IGKVDG was therefore not used in further experiments. Neither cathepsin S nor any of the peptides induced calcium responses in HeLa cells transfected with salmon sperm DNA alone. Cathepsin S and KVDGTS each activated NHEKs which endogenously express PAR2 (Figure 2b).

**Figure 3:**
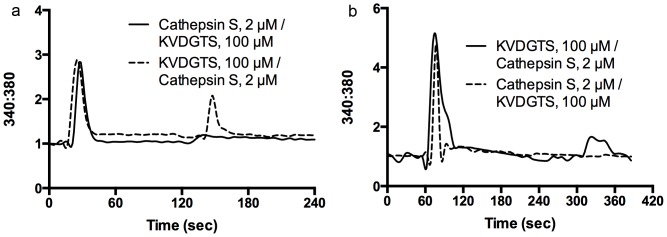
KVDGTS alters PAR2 response to cathepsin S. a) HeLa cells transfected with PAR2 cDNA were treated with KVDGTS and subsequently with cathepsin S after a 2 minute interval (dotted line). Pre-treatment with KVDGTS attenuates the response to cathepsin S. HeLa cells treated with cathepsin S failed to respond subsequently to KVDGTS (100 µM) (solid line). b) KVDGTS (100 µM) elicted calcium responses in NHEKs and the subsequent response to cathepsin S (2 µM) was attenuated. Treatment with cathepsin S abolishes the response to the subsequent addtion of KVDGTS. The second agent was delivered at the 300 second timepoint. KVDGTS (100 µM), cathepsin S (2 µM).

As hexapeptides activate the receptors without cleavage, we predicted that incubation with a hexapeptide followed by a protease would still elicit a signal, although perhaps diminished. To test this hypothesis, HeLa cells transfected with PAR2 were treated with KVDGTS and subsequently with cathepsin S after different time intervals (2, 3, 5, 10, 30 and 60 minutes). As predicted, following treatment with KVDGTS, calcium signals were present but reduced in response to cathepsin S at all of these timepoints. The data in Figure 3a represent the interval at 2 minutes and is representative of all timepoints examined. Similar results were found when KVDGTS was applied followed by SLIGRL, papain (2 µM) or trypsin (10 nM) (not shown). These studies were performed at room temperature and receptor turnover would be expected to be minimal.

In contrast, when cathepsin S, papain or trypsin were applied first, subsequent treatment with protease, KVDGTS or SLIGRL failed to induce a calcium response at any time up to 60 minutes after initial protease treatment. The data for cathepsin S followed by KVDGTS are shown in Figure 3a. Similar observations were made in sequential treatment studies in NHEKs. Pretreatment with KVDGTS diminished the subsequent response to cathespin S whereas pretreatment with cathepsin S abolished the subsequent response to KVDGTS (Figure 3b).

These experiments were performed at room temperature which would not allow for receptor turnover. It is likely that exposure of either HeLa cells or NHEKs to protease under these conditions resulted in clearance of intact receptors. This scenario may explain why subsequent incubation with peptides did not generate a response.

### PAR2 activation by KVDGTS stimulates second messenger activation

**Figure 4:**
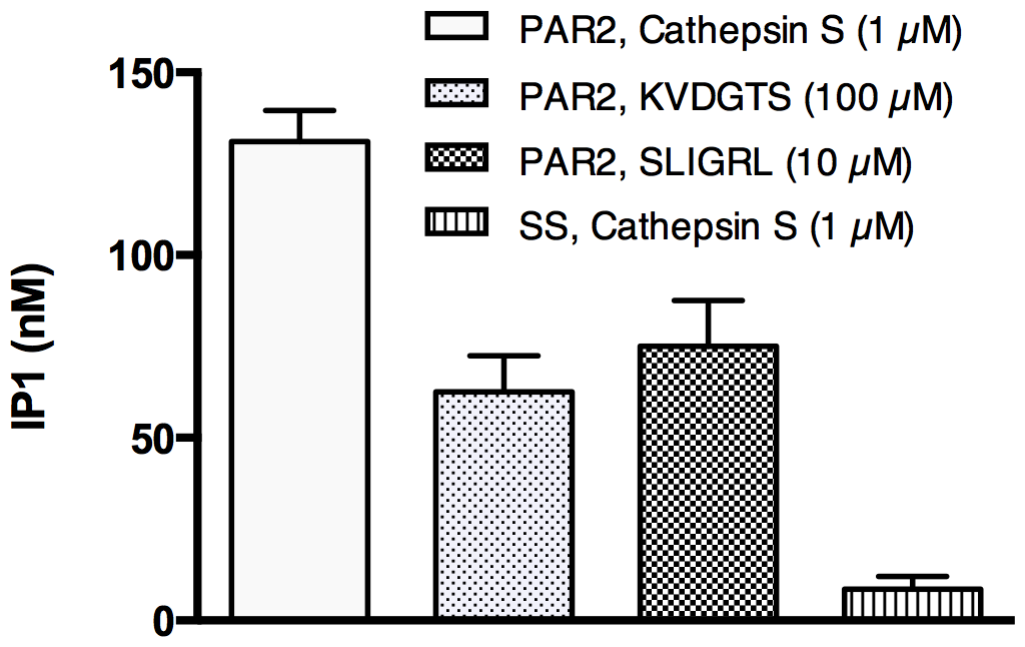
Cathepsin S and hexapeptide agonists increase the concentration of inositol phosphate. IP1 is a downstreatm metabolite of IP3. IP1 concentrations were measured following treatment with cathepsin S, KVDGTS and SLIGRL in HeLa cells that had been transfected with PAR2 cDNA. Hexapeptide agonists activated this signaling cascade downstream of PAR2, but to a lesser extent than cathepsin S. Cathepsin S had no effect above baseline IP1 levels on salmon sperm transfected cells. Cathepsin S (1 µM), KVDGTS (100 µM) and SLIGRL (10 µM).

To confirm that KVDGTS induces PLC activation and triggers the ionositol phosphate (IP) cascade similar to cathepsin S or other PAR2 agonists, we measured the generation of IP1, a downstream metabolite of IP3 that accumulates in cells following receptor activation. HeLa cells were transfected with PAR2 cDNA and IP1 levels were assayed following 1 hour of exposure to KVDGTS, SLIGRL or cathepsin S. Treatment with KVDGTS and SLIGRL produced similar levels of IP1, approximately 54 nM and 66 nM respectively (Figure 4) after subtracting baseline values. Cathepsin S treatment consistently generated higher levels of IP1 than either SLIGRL or KVDGTS, 122 nM in this particular experiment, a finding that was reproducible. This finding suggests that proteases and their associated tethered ligands provide more efficient activation of the receptor as compared to free hexapeptides. This result is consistent with the observation that high or pharmacologic concentrations of hexapeptides are needed to generate *in vitro* or *in vivo* responses as noted in the Discussion.

**Figure 5:**
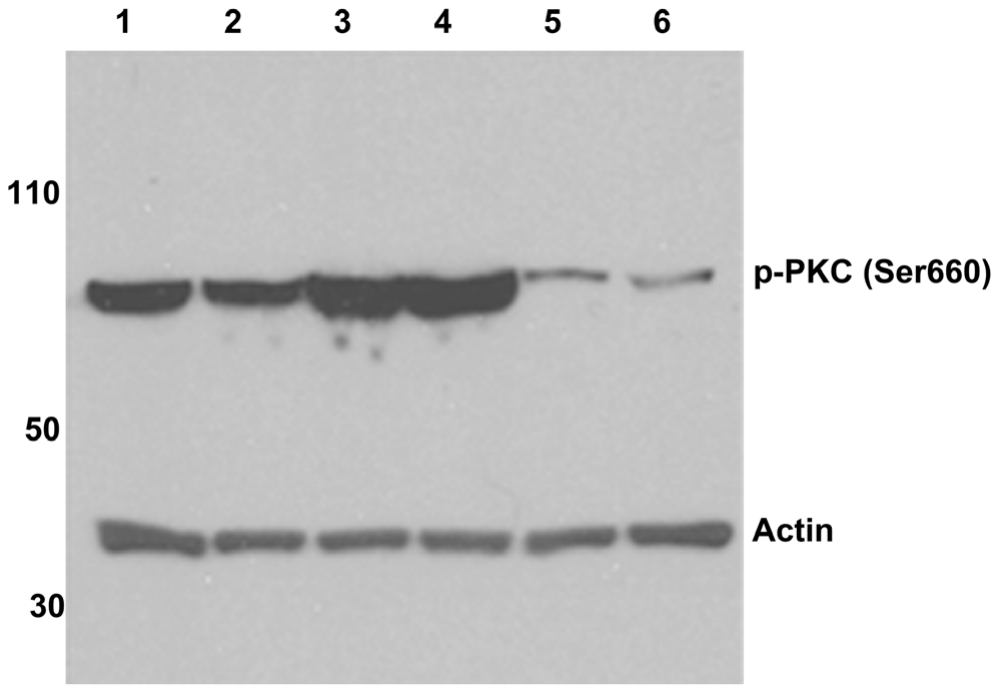
Cathepsin S and PAR2 peptide agonists induce PKC Ser660 phosphorylation in HeLa cells. The Western blot was performed on HeLa cells that had been transfected with PAR2 cDNA. Treatments included SLIGRL (lane 1), SLIGKVDGTS (lane 2), KVDGTS (lane 3) and cathepsin S (lane 4). Non-transfected HeLa cells treated with SLIGRL (10 µM) (lane 5) and PAR2-transfected but untreated HeLa cells (lane 6) served as controls. SLIGRL (10 µM), SLIGKVDGTS (10 µM), KVDGTS (100 µM) and cathepsin S (1 µM).

We next examined the capacity of KVDGTS to trigger downstream signaling cascades in HeLa cells, manifest by phosphorylation of protein kinase C (PKC). HeLa cells were treated with KVDGTS and Western blot analysis was performed to determine if KVDGTS stimulated PKC phosphorylation. KVDGTS caused an increase in the level of phosphorylation of PKC as compared to untreated controls (Figure 5). SLIGKVDGTS (10 µM), SLIGRL and cathepsin S (1 µM) were also included in this analysis. The detectable levels of phosphorylation were similar between KVDGTS and cathepsin S whereas SLIGRL and SLIGKVDGTS had a weaker effect. This finding was reproducible. This differential result suggests that KVDGTS and SLIGRL can elicit different downstream signals following the activation of PAR2.

### Cathepsin S fails to activate mutant PAR2 while tethered ligand hexapeptides retain activity

**Figure 6:**
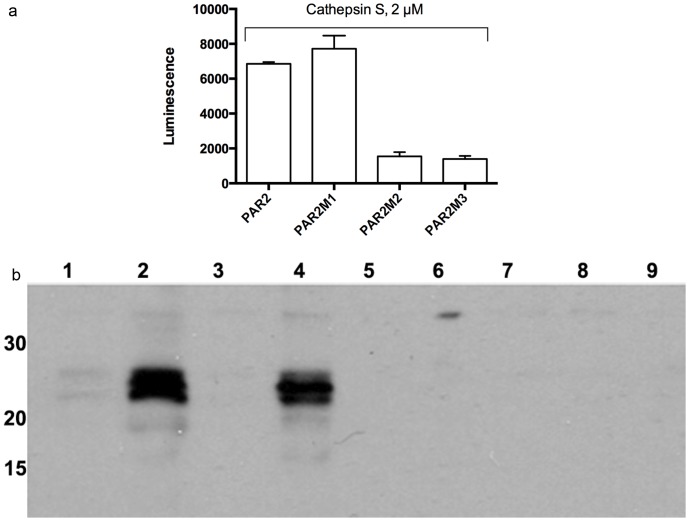
Cathepsin S cleavage of the PAR2 N-terminus requires glycine^40^. a) Luciferase luminescence was measured after cathepsin S (2 µM) treatment of HeLa cells expressing luciferase-PAR2 (bar 1), PAR2M1 (bar 2), PAR2M2 (bar 3), and PAR2M3 (bar 4). Luminescence of the transfected cells without cathepsin S treatment was subtracted from each of the measurements. b) Western blots were performed on supernatants of luciferase-PAR2 and PAR2 mutant-transfected HeLa cells following treatment with cathepsin S. Luciferase-PAR2, cathepsin S(−) (lane 1); luciferase-PAR2, cathepsin S (+) (lane 2); PAR2M1, cathepsin S (−) (Lane 3); PAR2M1 cathepsin S (+) (lane 4); PAR2M2, cathepsin S (−) (lane 5); PAR2M2, cathepsin S (+) (lane 6); PAR2M3, cathepsin S (−) (lane 7); PAR2M3, cathepsin S (+) (lane 8) and non-transfected HeLa cells, cathepsin S (+) (lane 9). Molecular weight markers on the left side of the blot are in kDa.

We next sought to determine the relevance of cathepsin S cleavage sites identified in the synthetic N-terminal PAR2 sequence as compared to the equivalent sites in the intact receptor. Selected point mutations were introduced near the N-terminus of full-length PAR2 to generate 3 distinct mutant receptors. The leucine^38^ residue at the cleavage site associated with IGKVDG was changed to alanine to generate PAR2M1. A second mutant receptor, PAR2M2, was generated in which the gylcine^40^ residue associated with KVDGTS was changed to alanine. A third mutant receptor, PAR2M3, was generated in which the leucine^38^ and glycine^40^ residues were each changed to alanines. We then introduced a *Gaussia* luciferase tag to the N-terminus of PAR2 and the mutant receptors. Luciferase-tagged receptors were transfected into HeLa cells followed by incubation with cathepsin S. Luciferase activity was assessed in the media (Figure 6a). While cathepsin S cleaved the native and PAR2M1 receptors, it did not cleave PAR2M2 or PAR2M3. This result highlights the importance of the glycine^40^ residue in receptor cleavage. A western blot using an anti-luiferase antibody confirmed the results of the luminescence assay (Figure 6b).

**Figure 7:**
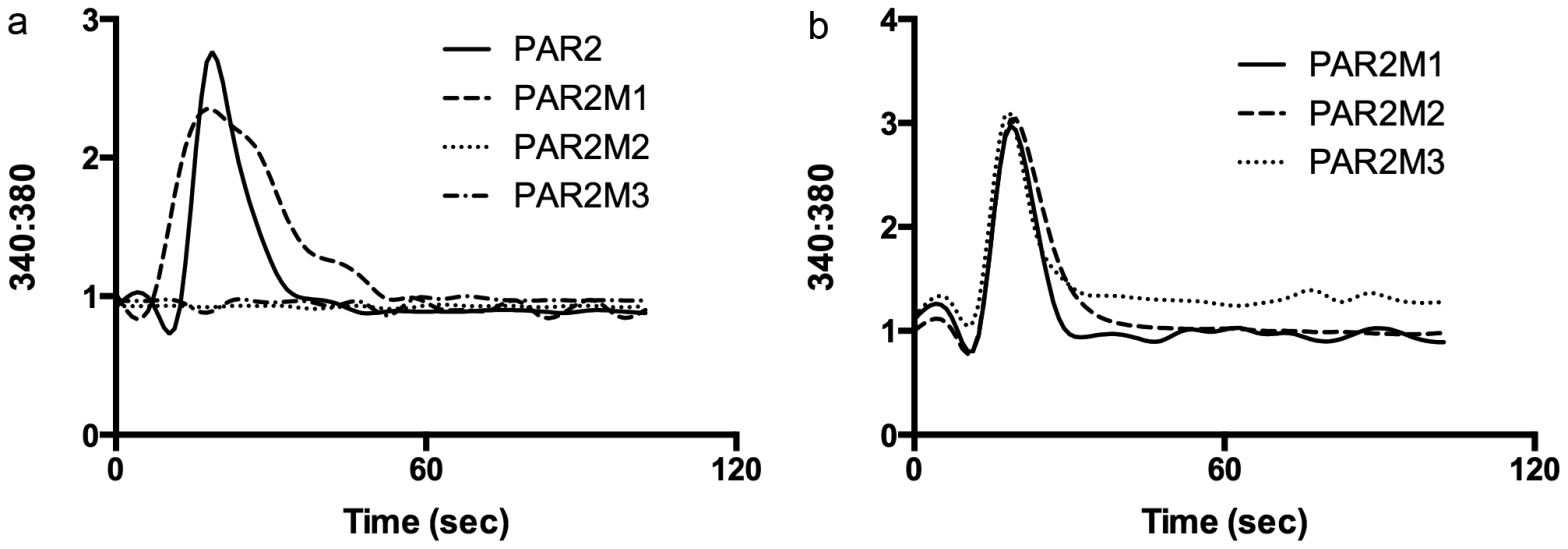
Cathepsin S fails to activate mutant PAR2 but KVDGTS retains activity on mutant PAR2. a) Calcium imaging was performed in HeLa cells transfected with native or mutant PAR2 receptors following treatment with cathepsin S. Cathepsin S (2 µM) was able to stimulate native PAR2 and PAR2M1 but not PAR2M2 or PAR2M3. b) In contrast, KVDGTS activated PAR2 and all three substitution mutants. Cathepsin S (2 µM), KVDGTS (100 µM).

To complement and confirm the luciferase studies, we used calcium imaging to investigate the effects of cathepsin S and KVDGTS on mutant receptors. Cathepsin S induced normal calcium responses in HeLa cells transfected with PAR2M1. Cathepsin S did not induce calcium responses in either the PAR2M2 or PAR2M3 mutants. These results support the critical importance of glycine^40^ for cleavage and activation by this protease (Figure 7a). KVDGTS activated all three mutant receptors with a calcium reponse curve similar to that of the wildtype PAR2 receptor (Figure 7b). These data reveal that KVDGTS activation of PAR2 does not depend on the cathepsin S cleavage sites.

## Discussion

Proteases and PARs have separately and together been implicated in the pathogenesis of pain, itch, asthma, cardiovascular disease and numerous other conditions associated with inflammation[4], [26]–[28]. Serine proteases had been considered the primary activators and inactivators of PAR2. However, recent publications support a role for cysteine proteases as well as other non-serine proteases in activating and regulating this receptor[5], [29]. We previously showed that cathepsin S, a cysteine protease, activates PAR2 but had not identified the mechanism by which activation occurred. In the present study, we show that the mechanism depends upon cleavage at a specific site near the N-terminus of PAR2. This cleavage site is distinct from that at which serine proteases cleave.

Treatment of the synthetic N-terminus of PAR2 with cathepsin S, followed by MS/MS sequencing, led us to evaluate two potential cathepsin S cleavage sites. These sites were separated by only two residues. Substitution mutants were generated at each site individually and together. The single substitution mutant closest to the N-terminus retained activity in response to cathepsin S. The single substitution mutant only two residues away was unresponsive to cathepsin S, as was the double mutant. Mutants that could not be cleaved by cathepsin S failed to generate downstream signaling. KVDGTS, the hexapeptide corresponding to the tethered ligand generated by cleavage at the distal site, activates wildtype and all three mutant PAR2 receptors. We conclude that cathepsin S cleavage of PAR2 generates a tethered ligand that activates the receptor. KVDGTS is distinct from SLIGRL, the conventional tethered ligand generated by serine proteases. SLIGRL is derived from the sequence of mouse PAR2 and the last two residues of the human equivalent, SLIGKV, are the first residues in KVDGTS. As reported here, the decapeptide SLIGKVDGTS, which encompasses SLIGKV and KVDGTS, had activity comparable to that of SLIGRL.

In this report, cathepsin S cleavage was most frequent after glycine followed by leucine. The KVDGTS sequence is preceded by LIG, placing G^40^ in the P1 postion and L^38^ in the P3 postion. The finding of cleavage after glycine is consistent with what is reported in the MEROPS database and a recent report on proteomic identification of protease cleavage sites (PICS)[25]. These reports reveal that cleavage after leucine at P3 is associated with cathepsin S, but less so than with glycine at P1. Correlations were otherwise weak between these reports and the cleavage sites identified here. Cathepsin S rarely cleaves after alanine according to the PICS report. We were able to take advantage of this observation and demonstrated the importance of the glycine^40^ residue to the activation of PAR2 by cathepsin S. Substitution of glycine^40^ or both glycine^40^ and Leucine^38^ with alanine prevented cathepsin S from cleaving or otherwise activating PAR2.

Proteases can activate PARs but it has been hypothesized that they can also inactivate them. Consistent with this hypothesis, several studies have demonstrated that some proteases are capable of ‘disarming’ PARs by cleaving at specific sites downstream of the tethered ligand, thereby preventing subsequent activation by other proteases such as thrombin or trypsin[30]–[33]. Serine proteases including trypsin, mast cell tryptase and tissue kallikreins 5, 6 and 14 cleave PAR2 primarily following arginine residues[25], [31]. As the cathepsin S cleavage sites identified here are downstream of the site associated with SLIGRL or SLIGKV, it is possible that cathepsin S-induced proteolysis of PAR2 may remove cleavage targets of these and other circulating proteases. Likewise, more distal cleavage by serine proteases could inhibit cathepsin S cleavage. Together, proteases, be they serine or cysteine, may function as both direct activators and as modulators of PAR2. The presence of inhibitors, such as cystatin, could provide an additonal degree of homeostasis.

Based on the sequences of cathepsin S-induced cleavage products of PAR2, we synthesized KVDGTS. KVDGTS was capable of stimulating intracellular signaling cascades and calcium mobilization downstream of PAR2 activation in keratinocytes as well as in a heterologous cell line. We note that the kinetics of the calcium transients were somewhat different between cathepsin S and the peptides. These results may reflect what has previously been described as ‘functional selectivity’ or ‘biased agonism’[29], [34] in which different proteases or synthetic peptide ligands elicit distinct signaling responses through the activation of the same PAR. Ligand activation of PARs is thought to induce conformational changes within transmembrane helices that expose cytoplasmic domains necessary for interaction with heterotrimeric G protein subunits, which then signal to various effectors to promote diverse cellular responses[35], [36]. Protease cleavage may result in other conformational changes that alter the exposure of cytoplasmic domains relevant to signal transduction as compared to activation by binding of hexapeptide ligands alone. Although cathepsin S was effective at a concentration of 1–2 µM in these studies, suggesting weaker activtity as compared to trypsin, it is possible that the technical challenges in producing recombinant cathepsin S may result in material that is less active than naturally produced cathepsin S. SLIGRL and KVDGTS needed to be present at substantially higher concentrations in order to demonstrate activity. It is not clear why KVDGTS was active at 100 µM whereas SLIGRL was active at 10 µM. We caution against attributing much significance to this observation, as it may reflect a pharmacologic rather than a physiologic phenomenon. For comparison, in studies of scratching in mice, SLIGRL is used as a positive control in mM concentrations[37].

We have identified a new peptide ligand generated by cathepsin S cleavage of PAR2. These data underscore the potential importance of a role for cysteine proteases in mediating physiologic or pathophysiologic conditions including inflammation via effects on PAR2 activation or inactivation. Our findings support a mechanism by which an N-terminal tethered ligand generated by cathepsin S cleavage might propagate PAR2 signaling in keratinocytes, or other target cells, surrounding a primary focus of inflammation. A possible scenario with respect to cutaneous inflammation could be as follows: IFN-γ, a mediator of inflammation, is a potent inducer of cathepsin S in keratinocytes[38], the production of which could lead to PAR2 activation followed by itch and pain.

The observation that pretreatment with KVDGTS attenuated responses to cathepsin S, and vice versa, raises the possibility that endogenously generated extracellular peptides may limit PAR activation in response to cathepsin S, or other proteases, in the setting of inflammation *in vivo*. The presence of N-terminal peptide fragments may influence downstream recruitment of second messengers due to biased agonism, triggering distinct cellular responses to different degrees or types of inflammation. While PAR cleavage by proteases such as trypsin may be important in regulating PAR-associated processes, it is possible that PAR cleavage by a number of proteases, including cysteine and serine proteases, may contribute to the complexity of receptor regulation. Zymogen activation of proteases, endogenous protease inhibitors, peptide activation, cleavage by several classes of proteases at distinct sites and differential activation of downstream signaling pathways may all participate in the process of PAR activation and modulation.
